# Preoperative Systemic Immune-Inflammatory Index Predicts Occult Nodal Disease in Clinically Node-Negative Intrahepatic Cholangiocarcinoma

**DOI:** 10.1245/s10434-025-17781-0

**Published:** 2025-07-09

**Authors:** Jun Kawashima, Miho Akabane, Mujtaba Khalil, Selamawit Woldesenbet, Odysseas P. Chatzipanagiotou, Yutaka Endo, Kota Sahara, François Cauchy, Federico Aucejo, Hugo P. Marques, Rita Lopes, Andreia Rodriguea, Tom Hugh, Feng Shen, Shishir K. Maithel, Bas Groot Koerkamp, Irinel Popescu, Minoru Kitago, Matthew J. Weiss, Guillaume Martel, Carlo Pulitano, Luca Aldrighetti, George Poultsides, Andrea Ruzzente, Todd W. Bauer, Ana Gleisner, Itaru Endo, Timothy M. Pawlik

**Affiliations:** 1 Department of Surgery, The Ohio State University Wexner Medical Center and James Comprehensive Cancer Center, Columbus, OH USA; 2 Department of Gastroenterological Surgery, Yokohama City University, Yokohama, Japan; 3 Department of Transplant Surgery, University of Rochester Medical Center, Rochester, NY USA; 4 Department of HPB Surgery and Liver Transplantation, Beaujon Hospital, Clichy, France; 5 Department of Hepato-pancreato-biliary & Liver Transplant Surgery, Cleveland Clinic Foundation, Digestive Diseases and Surgery Institute, Cleveland, OH USA; 6 Department of Surgery, Curry Cabral Hospital, Lisbon, Portugal; 7 Department of Surgery, The University of Sydney, Sydney, NSW Australia; 8 Department of Surgery, Eastern Hepatobiliary Surgery Hospital, Shanghai, China; 9 Division of Surgical Oncology, Winship Cancer Institution, Emory University, Atlanta, GA USA; 10 Department of Surgery, Erasmus University Medical Centre, Rotterdam, The Netherlands; 11 Department of Surgery, Fundeni Clinical Institute, Bucharest, Romania; 12 Department of Surgery, Keio University, Tokyo, Japan; 13 Department of Surgery, Cancer Institute, New Hyde Park, Northwell Health, NY USA; 14 Department of Surgery, University of Ottawa, Ottawa, ON Canada; 15 Department of Surgery, Royal Prince Alfred Hospital, Camperdown, NSW Australia; 16 Department of Surgery, San Raffaele Hospital, Milan, Italy; 17 Department of Surgery, Stanford University, Stanford, CA USA; 18 Division of General and Hepatobiliary Surgery, University of Verona, Verona, Italy; 19 Department of Surgery, University of Virginia, Charlottesville, VA USA; 20 Department of Surgery, University of Colorado Denver, Denver, CO USA

## Abstract

**Background:**

Accurate preoperative diagnosis of nodal status in intrahepatic cholangiocarcinoma (ICC) remains challenging. The objective of the current study was to determine if the systemic immune-inflammatory index (SII) was associated with occult nodal disease (OND) among cN0 patients undergoing resection for ICC.

**Methods:**

Patients who underwent curative resection for ICC were identified from an international multi-institutional database. A multivariable logistic regression model was used to assess the relationship between SII and OND.

**Results:**

Among 490 patients who underwent curative resection with lymph node dissection (LND) for cN0 ICC, 135 (27.6%) had OND. Among these individuals, high SII (≥738.4) was independently associated with OND (odds ratio [OR], 1.85, 95% confidence interval [CI], 1.18–2.92). This association was consistent even among patients with cT1aN0M0 disease (OR, 1.85; 95% CI, 1.19–2.88). Interestingly, among patients with high SII and N0/Nx disease, individuals whose total number of lymph nodes examined (TLNE) was fewer than six had worse 3-year recurrence-free survival (RFS) than patients with a TLNE of six or more (38.8% vs 74.0%; *p *= 0.002). In contrast, RFS did not differ among patients with low SII and N0/Nx disease (TLNE <6 [49.1%] vs ≥6 [62.4%]; *p* = 0.099).

**Conclusions:**

High SII was an independent predictor of OND, even among patients with early-stage disease, suggesting that incorporating SII into preoperative risk assessment may refine staging and guide treatment strategies including the need for neoadjuvant therapy as well as the extent and adequacy of LND.

**Supplementary Information:**

The online version contains supplementary material available at 10.1245/s10434-025-17781-0.

Intrahepatic cholangiocarcinoma (ICC) is a primary liver malignancy with a rising global incidence, accounting for 10% to 20% of all liver cancers^1,2^ Surgical resection remains the only curative-intent treatment option, but long-term outcomes are poor with 5-year overall survival (OS) ranging from 25% to 40% and recurrence rates as high as 70%.^3,4^

Among prognostic factors, lymph node metastasis (LNM) is one of the strongest predictors of poor survival, with a median OS of only 7 to 14 months among patients with metastatic nodal disease.^5–7^ Given the dismal prognosis associated with LNM, patients with nodal disease may benefit from neoadjuvant therapy rather than upfront surgery.^8^ Furthermore, recent guidelines suggest that patients with very early-stage ICC, including individuals with small solitary tumors and node-negative disease, may be candidates for liver transplantation.^3^ Therefore, accurate preoperative assessment of nodal status is critical to guiding treatment strategies.

Occult nodal disease (OND), defined as pathologically confirmed nodal metastasis in patients without clinical or radiographic evidence of LNM, has been increasingly recognized in certain malignancies, such as lung, breast, colorectal, and pancreatic cancer, with important prognostic implications.^9–12^ Whereas LNM is a well-established predictor of poor survival, OND remains insufficiently characterized in ICC. Current imaging methods, including computed tomography (CT), magnetic resonance imaging (MRI), and positron emission tomography (PET), have limited sensitivity to detect nodal disease preoperatively.^3,5^ As a result, a subset of patients undergoes surgery with the assumption of node-negative disease, only to have metastatic nodal disease noted on surgical pathology, highlighting the need for improved preoperative risk stratification.^5^ Because OND status can impact prognosis and treatment decisions, identifying reliable preoperative markers is essential to improve risk stratification and guide individualized management strategies.^12^

Systemic inflammation plays a pivotal role in tumor progression by promoting extracellular matrix degradation, immune evasion, and angiogenesis, all of which facilitate metastatic spread.^13^ Among inflammatory markers, the systemic immune-inflammatory index (SII) has been identified as a prognostic indicator in various cancers, including ICC.^14,15^ Previously, our group demonstrated that SII was associated with survival outcomes for ICC patients undergoing resection.^15^ Given its potential link to tumor aggressiveness, we hypothesized that OND in ICC patients may be associated with an underlying systemic inflammatory response, which could be reflected in peripheral blood markers. Therefore, the current study sought to characterize the prevalence and prognostic significance of OND in ICC patients, and to define the association between SII and OND using a large, multi-institutional, international database.

## METHODS

### Data Source and Patient Selection

Patients who underwent curative-intent liver resection for clinically node-negative ICC between 2000 and 2023 were identified from the International Intrahepatic Cholangiocarcinoma Study Group database.^16^ Patients were excluded if they had (1) macroscopically positive surgical margins (R2 resection), (2) missing data on key clinicodemographic characteristics such as lymph node dissection (LND), total number of lymph nodes examined (TLNE) or number of LNM, or (3) no follow-up data.

Preoperative node-negative status was assessed based on imaging studies, including ultrasound (US), CT, MRI, or PET/CT. Lymph nodes (LNs) were diagnosed as metastatic based on the following criteria: (1) a minimal diameter of 10 mm or larger, (2) a minimal diameter smaller than 10 mm but location near the tumor with a contrast pattern similar to that of the tumor, (3) evidence of extra-nodal invasion (e.g., fluffing), (4) or positive uptake on PET/CT.^17^ Given the retrospective and multi-institutional nature of the study, minor variations in the interpretation and application of these criteria may have occurred across institutions. Final classification of cN0 status was ultimately based on the clinical judgment of the attending radiologists and surgeons at each center, consistent with real-world practice. The study was approved by the institutional review boards of all the participating institutions.

### Variables and Outcomes

Patient demographic and clinicopathologic variables included age, sex, American Society of Anesthesiologist (ASA) classification, year of surgery (i.e., 2000–2010, 2011–2023), geographic region (i.e., Western countries, Eastern countries), cirrhosis, preoperative carbohydrate antigen 19-9 (CA19-9; i.e., ≤54 U/ml, >54 U/ml, unknown), preoperative SII, method of preoperative LN assessment (i.e., US only, CT only, MRI only, CT+MRI, PET-CT+CT/MRI, unknown), type of surgery (i.e., minor hepatectomy, major hepatectomy), LND, use of minimally invasive surgery (MIS), TLNE, tumor size, tumor number (i.e., single lesion, multiple lesions), T category based on the American Joint Committee on Cancer (AJCC) eighth edition,^18^ pathologic nodal disease (i.e., N0 [negative], N1 [positive], Nx [not examined]), surgical margin, major vascular invasion, microvascular invasion (MVI), morphologic subtype (i.e., mass-forming [MF], intraductal growth [IG], periductal infiltrating [PI], mass-forming plus periductal infiltrating [MF+PI]), tumor grade (i.e., well-, moderately, poorly differentiated; undifferentiated), perineural invasion (PNI), postoperative severe complication, and receipt of adjuvant chemotherapy.

The SII was calculated as the neutrophil-to-lymphocyte ratio multiplied by the platelet count.^14,15^ The optimal cutoff point was determined to be 738.4, corresponding to the threshold that maximized the Youden index in the receiver operating characteristic analysis for OND positivity among patients who underwent LND.^19^ To assess the internal stability of this cutoff, we performed 2000 bootstrap re-samples. Hepatectomy was classified as major (≥3 segments) or minor (≤2 segments).^20^ Severity of postoperative complications was defined according to the Clavien-Dindo classification system (grades I to V). Severe complications were defined as a Clavien-Dindo classification of III or higher.^21^

The primary outcome was OND, defined as pathologically confirmed nodal metastasis in patients without clinical or radiographic evidence of LN. The secondary outcome was recurrence-free survival (RFS), defined as the time elapsed between the date of liver resection and recurrence, either confirmed on biopsy or using evidence of a suspicious lesion on follow-up imaging.

### Statistical Analysis

Descriptive statistics are presented as median values with interquartile ranges (IQRs) for continuous variables and as frequencies with percentages for categorical variables. Continuous variables were compared using the Mann-Whitney *U* or Kruskal-Wallis test, as appropriate. Categorical variables were compared with the chi-square test or Fisher’s exact test. Multiple imputations with chain equations (MICE) procedures were used to handle missing values.^22^ Survival was estimated using the Kaplan-Meier method and log-rank tests.

Logistic regression analysis was used to assess the association between various clinicopathologic factors and OND among the patients who underwent LND. Several potential perioperative prognostic predictors, including SII, were selected based on clinical importance. Variables significantly associated with OND in the univariable analysis (*p* < 0.10) were subsequently included in the multivariable model. In addition, unadjusted restricted cubic splines with four pre-specified knots were generated to illustrate the relationship between the continuous SII and the log odds of OND.

In addition, to evaluate the association between SII and OND in early-stage ICC, uni- and multivariable logistic regression analyses also were performed among the patients with cT1aN0M0 ICC who underwent LND. The patients with cT1aN0M0 disease were defined as individuals who had a solitary tumor 5 cm or smaller in size without vascular invasion, evidence of preoperative nodal disease on imaging, involvement of local extrahepatic structures by direct invasion, or extrahepatic disease.^18^

To evaluate whether the impact of adequate LND, defined as LND with TLNE ≥6,^18^ on accurate staging and prognostic assessment was associated with SII, we analyzed RFS by comparing patients with TLNE <6 and those with TLNE ≥6 among all the pathologic N0 or Nx patients, regardless whether they underwent LND, stratified by SII.

Furthermore, to determine whether adjuvant chemotherapy modifies recurrence risk according to SII status among patients with TLNE <6, a multivariable Cox regression analysis was performed that included an interaction term between SII and adjuvant chemotherapy.

Statistical significance was set at an alpha of 0.05. All analyses were performed using R version 4.4.2 (R Project for Statistical Computing, Vienna, Austria).

## RESULTS

### Patient Demographics

Among the 1041 patients who met the inclusion criteria, 597 (57.3%) were male with a median age of 61 years (IQR, 53–69 years). Of the 1041 patients, 420 (40.3%) were classified as ASA >2, and 146 (14.0%) had cirrhosis. Preoperative laboratory data demonstrated that approximately one in four patients (*n* = 251, 24.1%) had an elevated CA19-9 level (> 54 U/ml). The median SII was 570.5 (IQR, 379.5–941.4), with 381 (36.6%) patients classified as having a high SII (≥ 738.4). To assess the robustness of this cutoff, a bootstrap analysis was performed using 2000 re-samples. The median bootstrapped cutoff was 729.6, with a 95% confidence interval (CI) ranging from 464.7 to 908.0. The original threshold of 738.4 fell well within this interval, supporting its stability.

Approximately half of the patients (*n* = 567, 54.5%) underwent major hepatectomy, whereas 490 (47.1%) underwent LND. A summary of the number and proportion of patients who underwent LND at each participating institution is provided in Table S1.

Regarding pathologic findings, the median tumor size was 5.8 cm (IQR, 4.0–8.0 cm), and 142 (13.6%) of the patients had multinodular lesions. Of the 1041 patients, 571 (54.9%) had pathologic T1 disease, and 135 (13.0%) had node-positive disease. Histologic examination showed major vascular invasion in 137 (13.2%) of the patients, whereas the examination showed MVI in 307 (29.5%) the patients, PI/MF+PI subtype in 120 (11.5%) of the patients, poorly or undifferentiated tumors in 162 (15.6%) of the patients, and PNI in 201 (19.3%) of the patients. Postoperatively, 182 (17.5%) of the patients experienced severe complications, and 318 (30.5%) received adjuvant chemotherapy (Table 1).

**Table 1:** Clinicopathologic characteristics of the analytic cohort

Characteristics	All patients
	(*n* = 1041) *n* (%)
Median age: years (IQR)	61 (53–69)
Male sex	597 (57.3)
ASA classification >2	420 (40.3)
Geographic region, Western countries	637 (61.2)
Year of surgery (2011–2023)	613 (58.9)
Cirrhosis	146 (14.0)
CA19-9 (U/ml)	
≤ 54	327 (31.4)
> 54	251 (24.1)
Unknown	463 (44.5)
Median SII (IQR)	570.5 (379.5–941.4)
Low (< 738.4)	660 (63.4)
High (≥ 738.4)	381 (36.6)
Method of preoperative lymph node assessment	
US only	34 (3.3)
CT only	495 (47.6)
MRI only	320 (30.7)
CT+MRI	68 (6.5)
PET-CT + CT/MRI	90 (8.6)
Unknown	34 (3.3)
Major hepatectomy	567 (54.5)
Lymph node dissection	490 (47.1)
Minimally invasive surgery	54 (5.2)
Median TLNE (IQR)	0 (0–3)
< 6	858 (82.4)
≥ 6	183 (17.6)
Median tumor size: cm (IQR)	5.8 (4.0–8.0)
≤ 5	447 (42.9)
> 5	594 (57.1)
Multiple lesions	142 (13.6)
Pathologic T category	
T1	571 (54.9)
T2	160 (15.4)
T3	282 (27.1)
T4	28 (2.7)
Pathologic N category	
N0	355 (34.1)
N1	135 (13.0)
Nx	551 (52.9)
Surgical margin, R1	153 (14.7)
Major vascular invasion	137 (13.2)
Microvascular invasion	307 (29.5)
Morphologic type, PI/MF+PI	120 (11.5)
Grade (poorly differentiated/undifferentiated)	162 (15.6)
Perineural invasion	201 (19.3)
Severe complication	182 (17.5)
Adjuvant chemotherapy	318 (30.5)

IQR, Interquartile range; ASA, American Society of Anesthesiologists; CA19-9, carbohydrate antigen 19-9; SII, Systemic immune-inflammatory index; US, ultrasound; CT, Computed tomography; MRI, Magnetic resonance imaging; PET-CT, Positron emission tomography-CT; TLNE, Total number of lymph nodes examined; PI/MF+PI, Periductal-infiltrating/mass-forming plus periductal-infiltrating

### Risk Factors for OND Among the Patients Who Underwent LND

**Fig. 1:**
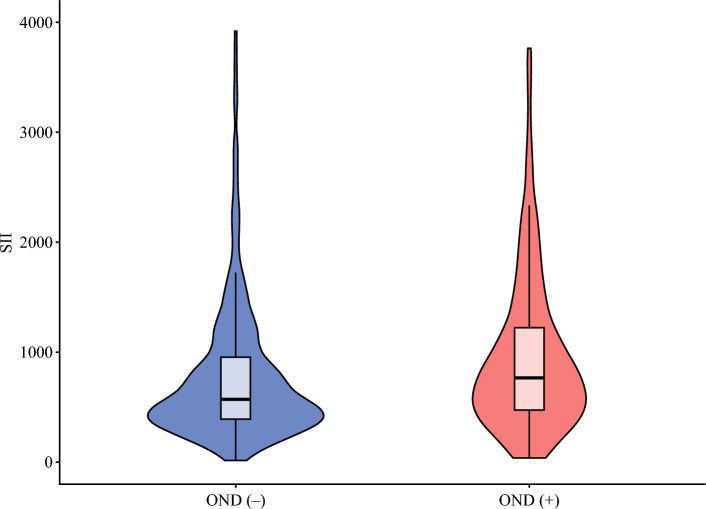
Violin plot illustrating the distribution of the systemic immune-inflammatory index (SII) based on the presence of occult nodal disease (OND) among patients who underwent lymph node dissection

Among the 490 patients who underwent LND, 135 (27.6%) exhibited OND. After a median follow-up period of 20.0 months (IQR, 9.7–39.9 months), the patients with OND had worse RFS than those with pN0 (3-year RFS: 22.0% [95% CI, 14.5–33.3] vs 47.8% [95% CI, 41.9–54.5; *p *< 0.001; Fig. S1). The patients with OND were more likely to have elevated CA19-9 levels (>54 U/ml: *n* = 57 [42.2%] vs *n* = 86 [24.2%]; *p* < 0.001) and higher SII (median, 791.9 [IQR, 480.5–1311.6] vs 605.4 [IQR, 395.1–1046.6]; *p* = 0.003; Fig. 1). In addition, OND was more frequently observed in the patients who underwent major hepatectomy (*n* = 114 [84.4%] vs *n* = 245 [69.0%]; *p* = 0.001), and in the patients with adequate LND (TLNE ≥6: *n* = 76 [56.3%] vs *n* = 107 [30.1%]; *p* < 0.001).

Pathologic analysis demonstrated that OND was more often associated with T3/4 disease (*n* = 76 [56.3] vs *n* = 119 [33.6]; *p* < 0.001), major vascular invasion (*n* = 46 [34.1] vs *n* = 45 [12.7]; *p* < 0.001), MVI (*n* = 78 [57.8] vs *n* = 122 [34.4]; *p* < 0.001), PI/MF+PI histologic subtype (*n* = 39 [28.9] vs *n* = 50 [14.1]; *p* < 0.001), poorly differentiated/undifferentiated tumors (*n* = 35 [25.9] vs *n* = 61 [17.2]; *p* = 0.040), and PNI (*n* = 73 [54.1] vs *n* = 77 [21.7]; *p* < 0.001). Furthermore, the patients with OND were more likely to receive adjuvant chemotherapy (*n* = 81 [60.0] vs *n* = 133 [37.5]; *p* < 0.001) (Table 2).

**Table 2:** Comparison of clinicopathologic characteristics between patients with pN0 and those with pN1 among patients who underwent lymph node dissection

Characteristics	OND (–)	OND (+)	*p* Value
	(*n* = 355) *n* (%)	(*n* = 135) *n* (%)	
Median age: years (IQR)	64 (55–71)	65 (56–72)	0.598
Male sex	173 (48.7)	69 (51.1)	0.712
ASA classification >2	156 (43.9)	58 (43.0)	0.925
Geographic region, Western countries	275 (77.5)	98 (72.6)	0.312
Year of surgery (2011–2023)	234 (65.9)	93 (68.9)	0.605
Cirrhosis	29 (8.2)	8 (5.9)	0.517
CA19-9 (U/ml)			< 0.001
≤54	133 (37.5)	40 (29.6)	
>54	86 (24.2)	57 (42.2)	
Unknown	136 (38.3)	38 (28.1)	
Median SII (IQR)	605.4 (395.1–1046.6)	791.9 (480.5–1311.6)	0.003
Low (< 738.4)	216 (60.8)	58 (43.0)	0.001
High (≥ 738.4)	139 (39.2)	77 (57.0)	
Method of preoperative LN assessment			0.152
US only	14 (4.2)	6 (4.4)	
CT only	176 (52.5)	84 (62.2)	
MRI only	86 (25.7)	22 (16.3)	
CT+MRI	29 (8.7)	8 (5.9)	
PET-CT + CT/MRI	30 (9.9)	15 (11.1)	
Unknown	20 (5.6)	0 (0.0)	
Major hepatectomy	245 (69.0)	114 (84.4)	0.001
Minimally invasive surgery	15 (4.2)	2 (1.5)	0.228
Median TLNE (IQR)	3 (1–6)	7 (3–10)	< 0.001
< 6	248 (69.9)	59 (43.7)	< 0.001
≥ 6	107 (30.1)	76 (56.3)	
Median tumor size: cm (IQR)	6.0 (4.0–8.2)	6.0 (4.5–9.0)	0.136
≤ 5	139 (39.2)	48 (35.6)	0.530
> 5	216 (60.8)	87 (64.4)	
Multiple lesions	48 (13.5)	28 (20.7)	0.067
Pathologic T category			< 0.001
T1	166 (46.8)	33 (24.4)	
T2	70 (19.7)	26 (19.3)	
T3	111 (31.3)	60 (44.4)	
T4	8 (2.3)	16 (11.9)	
Surgical margin, R1	61 (17.2)	31 (23.0)	0.182
Major vascular invasion	45 (12.7)	46 (34.1)	< 0.001
Microvascular invasion	122 (34.4)	78 (57.8)	< 0.001
Morphologic type, PI/MF+PI	50 (14.1)	39 (28.9)	< 0.001
Grade (poorly differentiated/undifferentiated)	61 (17.2)	35 (25.9)	0.040
Perineural invasion	77 (21.7)	73 (54.1)	< 0.001
Severe complication	74 (20.8)	40 (29.6)	0.053
Adjuvant chemotherapy	133 (37.5)	81 (60.0)	< 0.001

OND, Occult nodal disease; IQR, Interquartile range; ASA, American Society of Anesthesiologists; CA19-9, Carbohydrate antigen 19-9; SII, Systemic immune-inflammatory index; LN, Lymph node; US, Ultrasound; CT, Computed tomography; MRI, Magnetic resonance imaging; PET-CT, Positron emission tomography-CT; TLNE, Total number of lymph nodes examined; PI/MF+PI, Periductal-infiltrating/mass-forming plus periductal-infiltrating

**Fig. 2:**
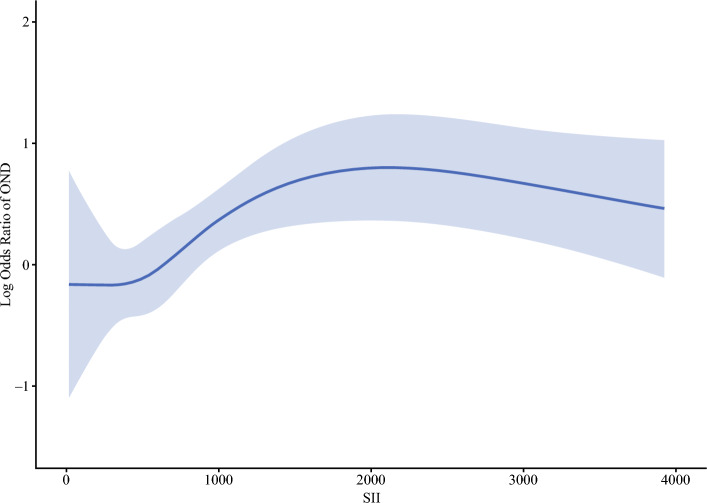
Restricted cubic spline plot illustrating the relationship between the continuous systemic immune-inflammatory index (SII) and the log odds of occult nodal disease (OND)

In the multivariable logistic regression analysis, after adjustment for relevant patient and preoperative tumor characteristics, high SII (odds ratio [OR], 1.85; 95% CI, 1.18–2.92; *p* = 0.008), TLNE ≥ 6 (OR, 2.42; 95% CI, 1.51–3.89; *p* < 0.001), major vascular invasion (OR, 2.31; 95% CI, 1.33–4.02; *p* = 0.003), and PNI (OR, 2.43; 95% CI, 1.49–3.95; *p* < 0.001) were each independently associated with OND (Table 3; Fig. S2). Interestingly, restricted cubic spline analysis demonstrated a non-linear association between SII and OND, with the log odds ratio for OND increasing as SII increased, from approximately 500 to 2000, followed by a plateau and a slight decline beyond 2500 (Fig. 2).

**Table 3:** Uni- and multivariable logistic regression analysis for occult nodal disease among patients who underwent lymph node dissection

	Univariable analysis		Multivariable analysis	
Variables	OR 95% CI	*p* Value	OR 95% CI	*p* Value
Age	1.00 (0.98–1.02)	0.869		
Male sex (ref: female)	1.10 (0.74–1.64)	0.638		
ASA classification >2 (ref: classification 1,2)	0.96 (0.64–1.43)	0.845		
Geographic region, Eastern countries (ref: Western countries)	1.30 (0.82–2.03)	0.259		
Year of surgery, 2011–2023 (ref: 2000–2010)	1.14 (0.75–1.76)	0.533		
Neoadjuvant chemotherapy	1.14 (0.59–2.13)	0.683		
Cirrhosis	0.71 (0.30–1.52)	0.403		
CA19-9 >54 U/ml (ref: ≤54 U/ml)	2.20 (1.36–3.60)	0.001	1.73 (0.98–3.05)	0.057
CA19-9 unknown (ref: ≤54 U/ml)	0.93 (0.56–1.54)	0.775	1.19 (0.66–2.14)	0.563
SII, high (≥738.4) (ref: low (<738.4)	2.06 (1.38–3.09)	< 0.001	1.85 (1.18–2.92)	0.008
Tumor size >5 cm (ref: ≤5 cm)	1.17 (0.78–1.77)	0.464		
Multiple lesions (ref: single lesion)	1.67 (0.99–2.79)	0.050	1.52 (0.82–2.77)	0.176
TLNE ≥6 (ref: <6)	2.99 (1.99–4.51)	< 0.001	2.42 (1.51–3.89)	< 0.001
Pathologic T category, T3/4 (ref: T1/2)	2.55 (1.71–3.84)	< 0.001	1.61 (0.99–2.61)	0.054
Major vascular invasion	3.56 (2.22–5.73)	< 0.001	2.31 (1.33–4.02)	0.003
Microvascular invasion	2.61 (1.75–3.93)	< 0.001	1.33 (0.82–2.16)	0.248
Morphologic type, PI/MF+PI (ref: MF/IG)	2.48 (1.53–3.99)	< 0.001	1.56 (0.90–2.67)	0.110
Grade, poorly differentiated/undifferentiated (ref: well/moderately)	1.69 (1.04–2.70)	0.030	1.34 (0.78–2.30)	0.283
Perineural invasion	4.25 (2.79–6.51)	< 0.001	2.43 (1.49–3.95)	< 0.001

OR, Odds ratio; CI, Confidence interval; ASA, American Society of Anesthesiologists; CA19-9, Carbohydrate antigen 19-9; SII, Systemic immune-inflammatory index; TLNE, Total number of lymph nodes examined; PI/MF+PI, periductal-infiltrating/ mass-forming plus periductal-infiltrating; MF, Mass-forming; IG, Intraductal growth

### Subgroup Analysis Among the cT1aN0M0 Patients Who Underwent LND

**Fig. 3:**
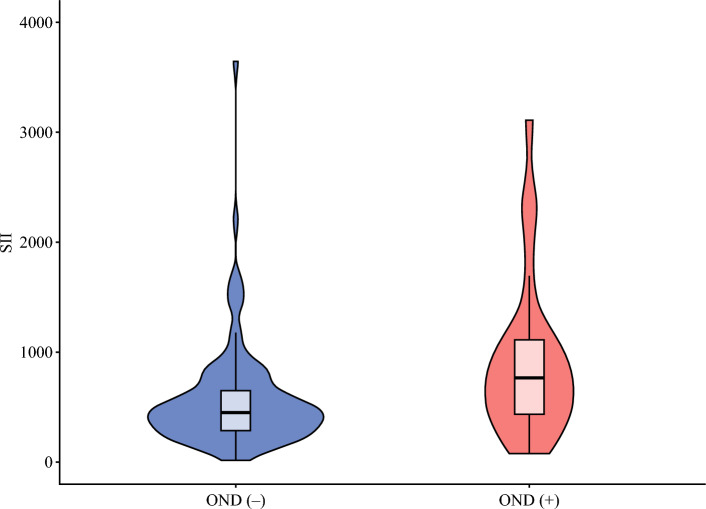
Violin plot illustrating the distribution of the systemic immune-inflammatory index (SII) based on the presence of occult nodal disease (OND) among patients who underwent lymph node dissection for cT1aN0M0 disease

Among 131 patients with cT1aN0M0 who underwent LND, 26 (19.8%) had OND. After a median follow-up period of 28.2 months (IQR, 10.7–45.7 months), the patients with OND had worse RFS than the individuals with pN0 (3-year RFS: 29.6% [95% CI, 13.9–63.1] vs 57.6% [95% CI, 47.4–69.9; *p* = 0.002; Fig. S3). The patients with OND also had higher SII (766.8 [IQR, 436.7–1112.4] vs 464.1 [IQR, 288.0–736.8]; *p* = 0.016; Fig. 3). In the multivariable logistic regression analysis among the patients with cT1aN0M0 who underwent LND, high SII (OR, 1.85; 95% CI, 1.19–2.88; *p* = 0.007) was an independent risk factor for OND. Additionally, OND was associated with TLNE ≥6 (OR, 2.43; 95% CI, 1.55–3.83; *p* < 0.001), MVI (OR, 1.71; 95% CI, 1.08–2.70; *p* = 0.022), PI/MF+PI subtype (OR, 1.70; 95% CI, 1.00–2.89; *p* = 0.049), and PNI (OR, 2.64; 95% CI, 1.65–4.23; *p* <0.001) (Table S2).

### Effect of Adequate Lymph Node Dissection on Accurate Staging: A Stratified Analysis by SII

**Fig. 4:**
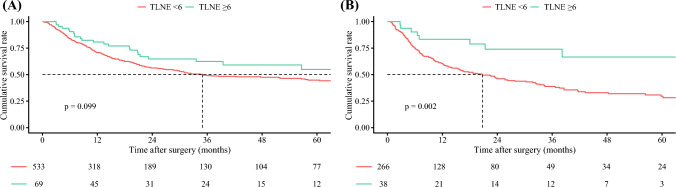
Kaplan-Meier curves for recurrence-free survival comparing pN0/Nx patients with the total number of lymph nodes examined (TLNE: <6 vs ≥6) stratified by **A** low systemic immune-inflammatory index (SII) and **B** high SII

In the entire cohort, 490 (47.1%) of the patients underwent LND, whereas 551 (52.9%) did not. A comparison of baseline characteristics stratified by LND status is detailed in Table S3. Notably, 183 (17.6%) of the patients had at least six LNs evaluated. Among the 602 (57.8%) patients with low SII and N0/Nx disease, the 3-year RFS did not differ between the patients with TLNE <6 and those with TLNE ≥6 (49.1% [95% CI, 44.3–54.3] vs 62.4% [95% CI, 50.6–76.9; *p* = 0.099). In contrast, among the 304 (29.2%) patients with high SII and N0/Nx disease, those with TLNE <6 had worse 3-year RFS than those with TLNE ≥6 (38.8% [95% CI, 32.5–46.4] vs 74.0% [95% CI, 58.8–93.2; *p* = 0.002; Fig. 4). In the multivariable Cox regression analysis of the patients with TLNE <6, including an interaction term between SII and adjuvant chemotherapy, no significant interaction was observed, suggesting that the effect of adjuvant chemotherapy on RFS did not meaningfully differ by SII status (Table S4).

## DISCUSSION

Curative-intent liver resection followed by adjuvant chemotherapy remains the cornerstone of treatment for patients with ICC.^3^ However, postoperative disease recurrence occurs in 50% to 70% of cases, limiting long-term survival and highlighting the need for improved risk stratification.^3,23^ In particular, patients with nodal disease face a dismal prognosis, with a median OS of only 7 to 14 months, even after curative resection.^5–7^ Given these poor outcomes, optimizing preoperative staging and surgical decision-making is critical.^5^

Despite its prognostic significance, accurate preoperative detection of nodal disease remains challenging in ICC.^5,24,25^ For instance, two systematic reviews and meta-analyses evaluating the role of preoperative PET for cholangiocarcinoma reported that the sensitivity to detect LNM ranged from only 37% to 64%.^24,25^ Consequently, a subset of patients is treated with surgery under the assumption of node-negative disease, only to have nodal metastasis on final pathology. This discrepancy highlights the need for reliable preoperative markers to predict LNM and guide appropriate surgical planning.

In this context, the current study is important because it demonstrated that patients with high SII were more likely to have OND, even after adjustment for clinicopathologic confounders. This association was consistent even among the early-stage ICC patients, including the patients with cT1aN0M0 disease, reinforcing SII as a robust predictive marker. Furthermore, among the patients with high SII and pathologic N0 or Nx disease, those with TLNE <6 had worse RFS than the patients with TLNE ≥6. These findings suggest that adequate LND is crucial for accurate staging and prognosis assessment, particularly for patients with high SII.

Cancer and inflammation are intricately linked, with the tumor immune microenvironment (TME) playing a central role in modulating tumor progression.^13,26^ Chronic inflammation promotes carcinogenesis through persistent immune activation, the release of reactive oxygen species, and direct cellular damage, leading to genetic alterations that drive tumor initiation and progression.^13,26^ Moreover, inflammatory cells within the TME, including neutrophils, macrophages, and dendritic cells, secrete cytokines, chemokines, and growth factors that facilitate angiogenesis, immune evasion, and metastatic spread.^26–28^ These pro-inflammatory signals activate key oncogenic pathways, such as NF-κB, STAT-3, and HIF-1α, further sustaining a tumor-supportive inflammatory milieu.^26–28^

Given this complex interplay between inflammation and cancer, systemic inflammatory markers have been widely investigated as prognostic and predictive biomarkers across various malignancies.^14,15,28–31^ Among these markers, SII has been identified as a prognostic factor in ICC.^15,31^ Previously, our group demonstrated that patients with high preoperative SII had a 70% higher risk of all-cause mortality and a 55% higher risk of cancer-specific death than those with low SII, suggesting that SII may serve as a more robust predictor of long-term outcomes than other inflammatory markers.^31^ These findings highlight the clinical relevance of SII as a biomarker for survival outcomes in ICC. More recently, studies in lung cancer and endometrial cancer have reported an association between elevated SII and LNM, suggesting that SII may serve as a predictive marker for nodal disease.^28,32^ In light of these findings, data in the current study further support the role of SII as a potential marker for OND in ICC.

The role of multidisciplinary treatment for resectable ICC has gained increasing attention.^33^ Instead of defaulting to upfront surgery, preoperative systemic therapy plays a critical role in identifying and stratifying patients based on their suitability for surgery.^16,33^ Given the aggressive nature of node-positive ICC, a paradigm shift toward preoperative therapy warrants consideration. Indeed, several retrospective studies have demonstrated the survival benefit of NAC for ICC patients with node-positive disease.^8,34^ Furthermore, a recent clinical trial evaluating neoadjuvant therapy for ICC has included clinically node-positive patients as a high-risk cohort.^35^

Liver transplantation has emerged as a promising curative option, particularly for early-stage ICC.^3^ Several studies have reported favorable outcomes, with 5-year OS ranging from 61% to 73%, leading to its recommendation in recent guidelines.^3,36–39^ The definition of early-stage disease varies across reports, but at the very least, clinical N0 patients with small solitary tumors have been suggested to benefit from liver transplantation. These findings highlight the importance of accurate preoperative staging for optimal treatment decision-making.

The current study demonstrated that patients with high SII were 85% more likely to have OND than individuals with low SII among patients with an initial diagnosis of node-negative disease. Notably, this finding was consistent even among the early-stage ICC patients. Importantly, SII is a non-invasive and easily measurable preoperative metric, making it a practical tool for risk stratification. Even among patients classified as cN0 by standard imaging methods (CT, MRI, PET-CT), additional diagnostic assessments may be warranted to refine staging and guide treatment selection. Recent studies have highlighted the potential role of endoscopic ultrasound (EUS) with fine-needle aspiration (FNA) to detect LNM.^3,40^ A retrospective study reported unsuspected LNM in 17% of ICC patients using EUS-guided sampling.^40^

Given these findings, integrating SII into preoperative decision-making may complement existing staging methods and help to refine patient selection for additional diagnostic interventions such as EUS-guided LN sampling. To illustrate this proposed clinical pathway, we developed an algorithm integrating SII status into nodal assessment and treatment planning (Fig. S4). Future studies should explore the combined utility of SII and EUS in identifying high-risk patients, ultimately improving treatment allocation and outcomes in ICC.

Routine LND to ensure accurate staging and prognosis assessment is recommended by several guidelines.^3^ In particular, the AJCC eighth edition of ICC staging recommends the retrieval of at least six LNs for complete nodal staging.^18^ To this point, data in the current study demonstrated that examining more than six LNs provided the greatest discriminatory power to identify true nodal involvement and predict long-term outcomes, further supporting the AJCC criteria. Interestingly, the current study showed that among the patients with high SII, those with TLNE <6 and pathologic N0/Nx had worse RFS than the patients with TLNE ≥6 and pathologic N0 disease. In contrast, among the patients with low SII and pathologic N0/Nx, RFS did not differ based on TLNE.

These findings suggest that for patients with high SII, which is considered an independent risk factor for OND, inadequate LN evaluation may result in underestimation of disease burden. As a consequence, a subset of patients classified as pathologic N0/Nx with TLNE <6 may, in fact, harbor residual nodal metastases that were not detected, leading to inaccurate staging and suboptimal treatment decisions. These data highlight the need for sufficient LN evaluation, particularly among patients with high SII, to improve staging accuracy and optimize oncologic outcomes.

Several limitations of the current study should be acknowledged when the findings are interpreted. As a retrospective analysis, there may have been residual selection bias. In addition, although the inclusion of multiple centers was a strength, the treatment strategies may have had some variability across different institutions. Specifically, differences in surgical techniques and the management of patients pre- and postoperatively may have contributed to variability in outcomes.

In addition, the definition of OND relied on pathologic examination of resected specimens, which may have been influenced by differences in the extent of LND performed across centers. Notably, a proportion of patients did not undergo LND, limiting our ability to fully assess the true prevalence of OND.

Furthermore, although SII emerged as a predictor of OND, its optimal cutoff value was determined based on the Youden index in this cohort, and further validation in prospective studies is necessary to establish standardized thresholds. Moreover, although the SII values used in this study were generally obtained from laboratory data collected immediately before surgery, the exact timing and clinical context of blood sampling (e.g., presence of cholangitis or other acute inflammatory conditions) were not available in the dataset. As such, unmeasured preoperative confounders may have affected the SII values. Additionally, data on the use of preoperative lymph node-sampling (e.g., EUS-FNA) were not available, precluding any assessment of concordance between preoperative findings and postoperative nodal pathology.

In conclusion, approximately one in four patients who underwent LND for clinically node-negative ICC had OND, which was associated with worse RFS. Notably, a high preoperative SII was an independent predictor of OND, even in the patients with early-stage disease. Furthermore, among the patients with high SII and pathologic N0 or Nx disease, inadequate LND (TLNE <6) was associated with significantly worse survival, highlighting the importance of adequate nodal evaluation in this high-risk population. Given the limited sensitivity of current imaging methods in detecting nodal metastasis, incorporating SII into preoperative risk assessment may aid in refining staging and guiding treatment strategies, such as the selection of candidates for additional diagnostic evaluation or consideration of neoadjuvant therapy.

## Supplementary Information

Below is the link to the electronic supplementary material.

Supplementary file1 (DOCX 1341 KB)

## Data Availability

Study data are not publicly available as they contain patient-level personal information but are available from the corresponding author on reasonable request.
