# Supplementary material for: Preoperative Systemic Immune-Inflammatory Index Predicts Occult Nodal Disease in Clinically Node-Negative Intrahepatic Cholangiocarcinoma
Source: Ann Surg Oncol. 2025 Jul 9;32(10):7152–62. doi: 10.1245/s10434-025-17781-0 (PMC12454466; doi:10.1245/s10434-025-17781-0)
Supplement: Supplementary file 1 — Supplementary file1 (DOCX 1341 KB) [file 10434_2025_17781_MOESM1_ESM.docx]

**Supplementary Figure 1**: Kaplan-Meier curves for recurrence-free survival comparing patients with occult nodal disease (OND) and those without OND among patients who underwent lymph node dissection


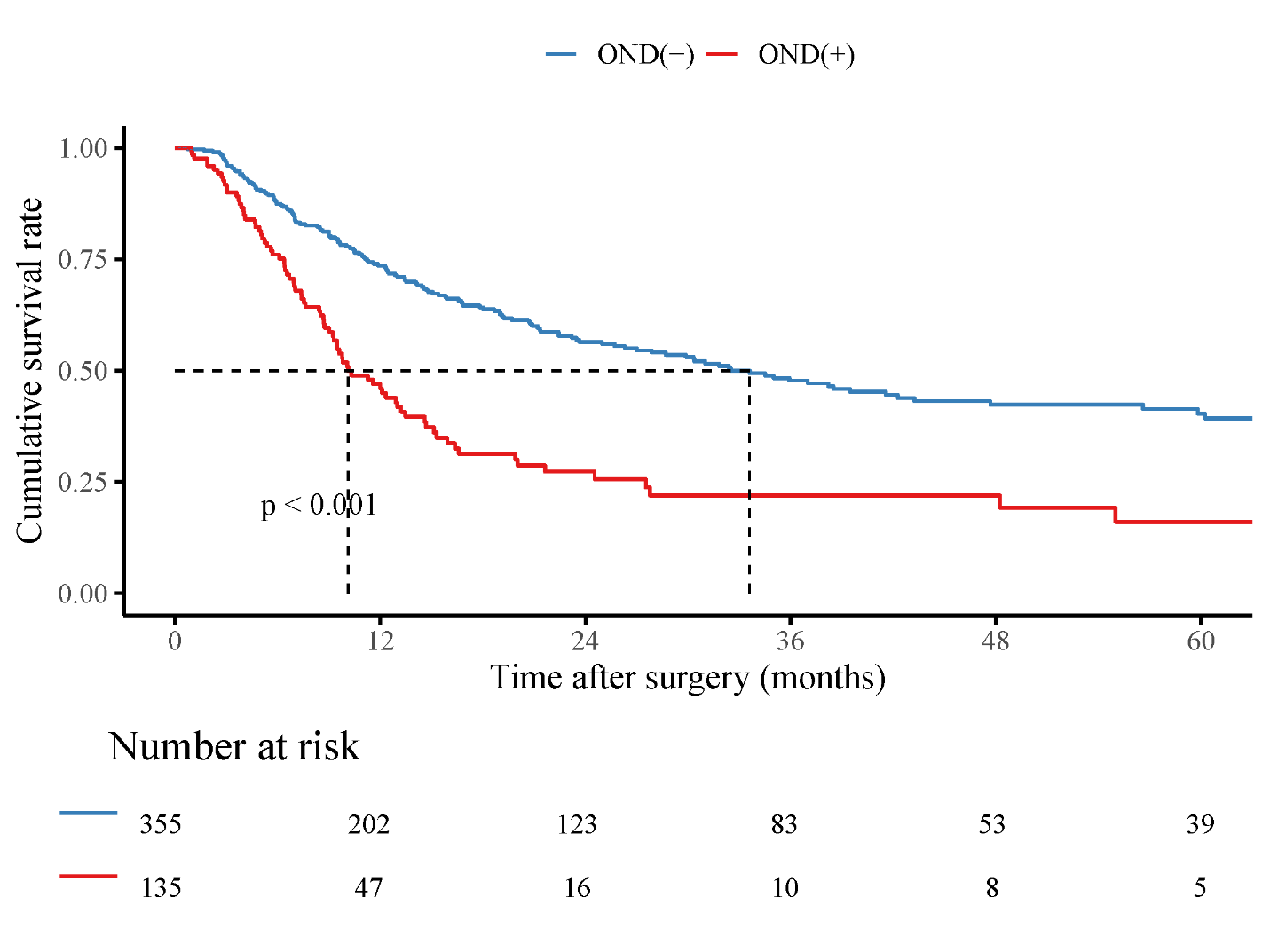


**Supplementary Figure 2**: A forest plot of independent predictors of OND

Abbreviations: **CA19-9,** carbohydrate antigen 19-9; **SII,** systemic immune-inflammatory index; **PI/MF+PI,** periductal infiltrating/ mass forming plus periductal infiltrating


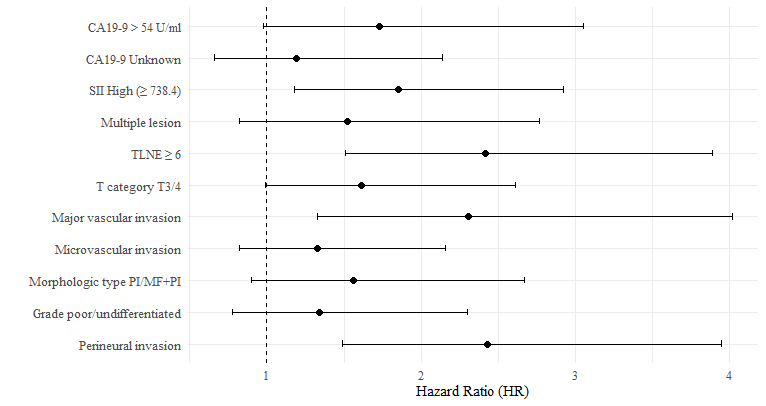


**Supplementary Figure 3**: Kaplan-Meier curves for recurrence-free survival comparing patients with occult nodal disease (OND) and those without OND among patients who underwent lymph node dissection for cT1aN0M0 disease


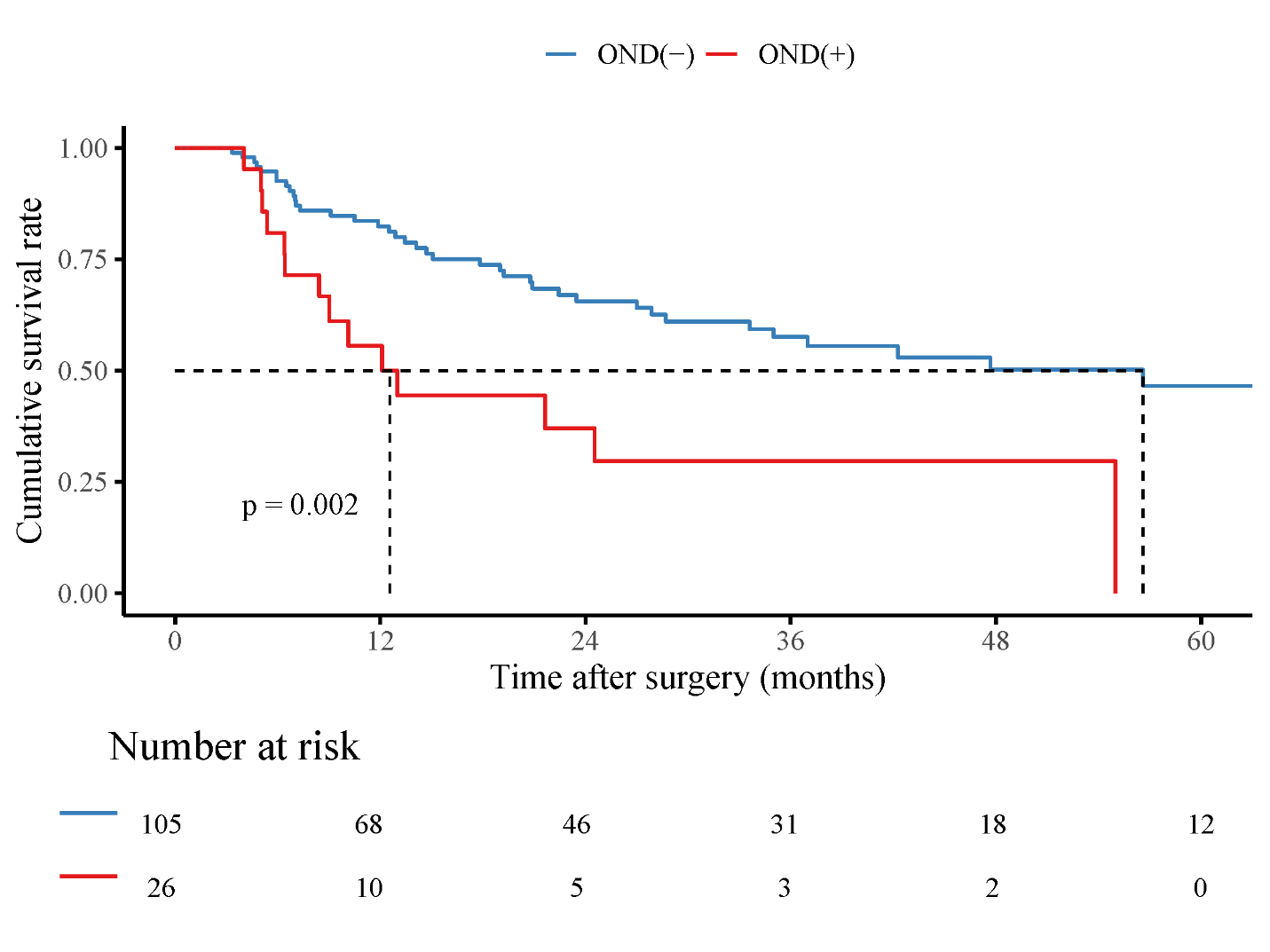


**Supplementary Figure 4**: A proposed algorithm integrating SII status into nodal assessment and treatment planning

Abbreviations: **SII,** systemic immune-inflammatory index; **CT,** computed tomography; **MRI,** magnetic resonance imaging; **PET-CT,** positron emission tomography-CT; **EUS-FNA,** endoscopic ultrasound-fine needle aspiration; **NAT,** neoadjuvant therapy


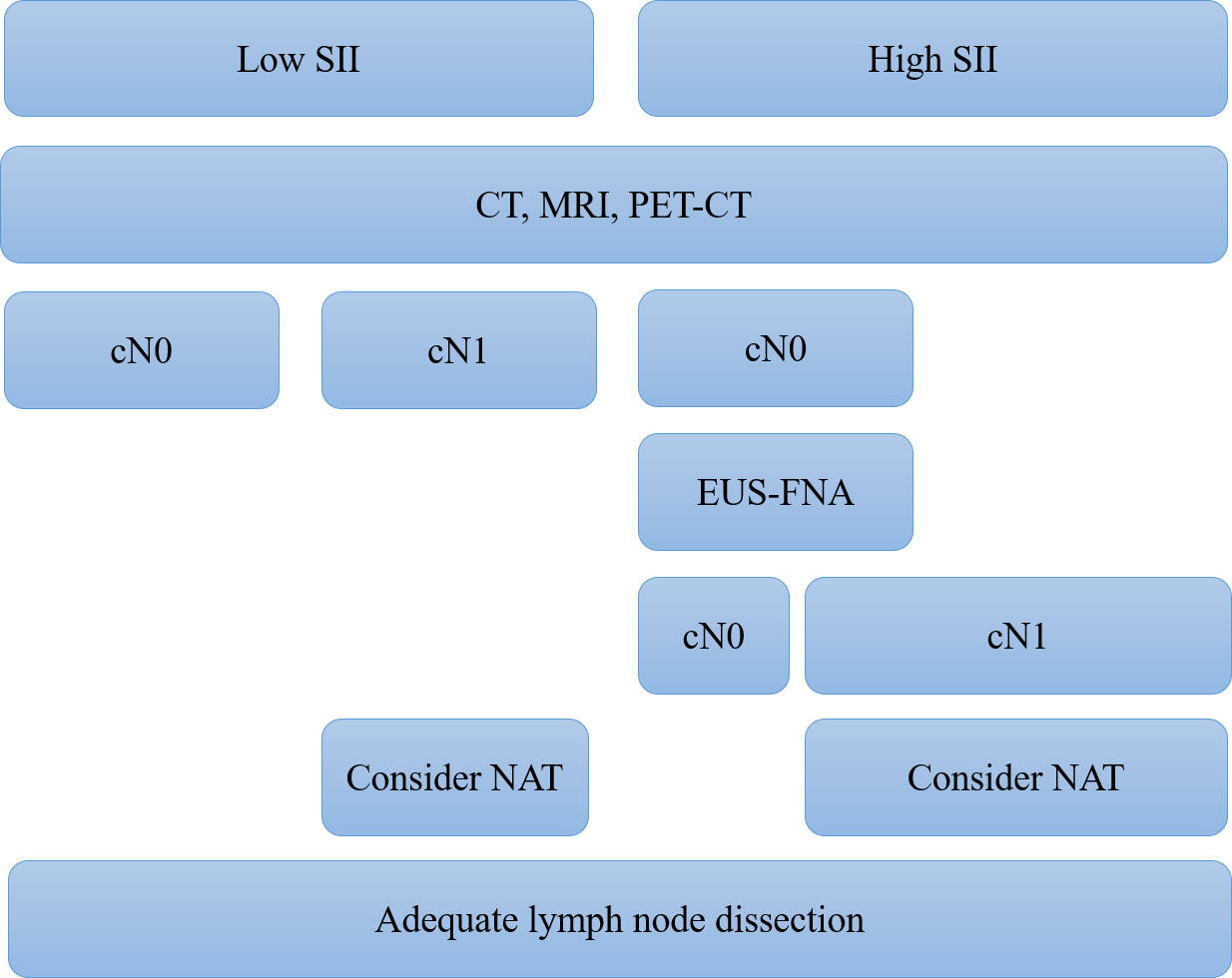


**Supplementary Table 1**. Lymph node dissection (LND) rates by institution.

|  | Total patients | LND cases (%) |
| --- | --- | --- |
| Institution 1 | 69 | 28 (40.6) |
| Institution 2 | 48 | 41 (85.4) |
| Institution 3 | 56 | 53 (94.6) |
| Institution 4 | 77 | 63 (81.8) |
| Institution 5 | 326 | 56 (17.2) |
| Institution 6 | 30 | 18 (60.0) |
| Institution 7 | 121 | 43 (35.5) |
| Institution 8 | 41 | 26 (63.4) |
| Institution 9 | 30 | 20 (66.7) |
| Institution 10 | 48 | 20 (41.7) |
| Institution 11 | 36 | 8 (22.2) |
| Institution 12 | 67 | 57 (85.1) |
| Institution 13 | 33 | 18 (54.5) |
| Institution 14 | 59 | 39 (66.1) |

**Supplementary Table 2**. Univariable and multivariable logistic regression analysis for occult nodal disease among patients who underwent lymph node dissection for cT1aN0M0 disease.

|  | Univariable analysis | |  | Multivariable analysis | |
| --- | --- | --- | --- | --- | --- |
| Variables | OR 95%CI | p-value |  | OR 95%CI | p-value |
| Age | 1.01 [0.97, 1.05] | 0.739 |  |  |  |
| Sex, male  (ref: Female) | 0.72 [0.30, 1.72] | 0.457 |  |  |  |
| ASA classification, > 2  (ref: Classification 1,2) | 1.44 [0.61, 3.46] | 0.407 |  |  |  |
| Geographic region, Eastern countries  (ref: Western countries) | 1.28 [0.48, 3.22] | 0.603 |  |  |  |
| Year of surgery, 2011-2023  (ref: 2000-2010) | 0.55 [0.23, 1.40] | 0.200 |  |  |  |
| Neoadjuvant chemotherapy | 0.38 [0.02, 2.13] | 0.367 |  |  |  |
| Cirrhosis | 0.41 [0.06, 1.53] | 0.247 |  |  |  |
| CA19-9, > 54 U/ml  (ref: ≤ 54 U/ml) | 7.47 [2.54, 24.43] | < 0.001 |  | 1.72 [1.00, 2.98] | 0.051 |
| CA19-9, Unknown  (ref: ≤ 54 U/ml) | 1.14 [0.33, 3.91] | 0.828 |  | 1.22 [0.70, 2.14] | 0.486 |
| SII, High (≥ 738.4)  (ref: Low [< 738.4]) | 3.54 [1.46, 8.77] | 0.005 |  | 1.85 [1.19, 2.88] | 0.007 |
| TLNE, ≥ 6  (ref: < 6) | 3.07 [1.28, 7.66] | 0.013 |  | 2.43 [1.55, 3.83] | < 0.001 |
| Microvascular invasion | 2.14 [0.88, 5.18] | 0.089 |  | 1.71 [1.08, 2.70] | 0.022 |
| Morphologic type, PI/MF+PI  (ref: MF/IG) | 4.84 [1.78, 13.21] | 0.002 |  | 1.70 [1.00, 2.89] | 0.049 |
| Grade, poor/undifferentiated,  (ref: well/moderate) | 2.15 [0.43, 8.82] | 0.303 |  |  |  |
| Perineural invasion | 4.53 [1.81, 11.46] | 0.001 |  | 2.64 [1.65, 4.23] | < 0.001 |

Abbreviations: **ASA,** American society of Anesthesiologists; **CA19-9,** carbohydrate antigen 19-9; **SII,** systemic immune-inflammatory index; **TLNE,** the total number of lymph nodes examined; **MF,** mass-forming; **IG,** intraductal growth; **PI/MF+PI,** periductal infiltrating/ mass forming plus periductal infiltrating

**Supplementary Table 3**. Comparison of clinicopathological characteristics stratified by LND.

| Characteristics | LND (-) | LND (+) | p-value |
| --- | --- | --- | --- |
|  | n = 551 | n = 490 |  |
| Age, years, median (IQR) | 58 [50, 67] | 64 [55, 71] | < 0.001 |
| Sex, male | 355 (64.4) | 242 (49.4) | < 0.001 |
| ASA classification, > 2 | 206 (37.4) | 214 (43.7) | 0.045 |
| Geographic region, western countries | 264 (47.9) | 373 (76.1) | < 0.001 |
| Year of surgery, 2011-2023 | 286 (51.9) | 327 (66.7) | < 0.001 |
| Cirrhosis | 109 (19.8) | 37 (7.6) | < 0.001 |
| CA19-9 |  |  | < 0.001 |
| ≤ 54 U/ml | 154 (27.9) | 173 (35.3) |  |
| > 54 U/ml | 108 (19.6) | 143 (29.2) |  |
| Unknown | 289 (52.5) | 174 (35.5) |  |
| SII, median (IQR) | 523.1 [356.7, 825.2] | 639.2 [414.7, 1122.2] | < 0.001 |
| Low (< 738.4) | 386 (70.1) | 274 (55.9) | < 0.001 |
| High (≥ 738.4) | 165 (29.9) | 216 (44.1) |  |
| Major hepatectomy | 208 (37.7) | 359 (73.3) | < 0.001 |
| Minimally invasive surgery | 37 (6.7) | 17 (3.5) | 0.027 |
| TLNE, median (IQR) | - | 3 [1, 8] | < 0.001 |
| < 6 | 551 (100.0) | 307 (62.7) | < 0.001 |
| ≥ 6 | - | 183 (37.3) |  |
| Tumor size, median (IQR) | 5.4 [3.9 7.6] | 6 [4, 8.5] | 0.003 |
| ≤ 5cm | 260 (47.2) | 187 (38.2) | 0.004 |
| > 5cm | 291 (52.8) | 303 (61.8) |  |
| Multiple lesions | 66 (12.0) | 76 (15.5) | 0.117 |
| Pathological T category |  |  | < 0.001 |
| T1 | 372 (67.5) | 199 (40.6) |  |
| T2 | 64 (11.6) | 96 (19.6) |  |
| T3 | 111 (20.1) | 171 (34.9) |  |
| T4 | 4 (0.7) | 24 (4.9) |  |
| Pathological N category |  |  |  |
| N0 | - | 355 (72.4) |  |
| N1 | - | 135 (27.6) |  |
| Nx | 551 (100.0) | - | < 0.001 |
| Surgical margin, R1 | 61 (11.1) | 92 (18.8) | 0.001 |
| Major vascular invasion | 46 (8.3) | 91 (18.6) | < 0.001 |
| Microvascular invasion | 107 (19.4) | 200 (40.8) | < 0.001 |
| Morphologic type, PI/MF+PI | 31 (5.6) | 89 (18.2) | < 0.001 |
| Grade, poor/undifferentiated | 66 (12.0) | 96 (19.6) | 0.001 |
| Perineural invasion | 51 (9.3) | 150 (30.6) | < 0.001 |
| Severe complication | 68 (12.3) | 114 (23.3) | < 0.001 |
| Adjuvant chemotherapy | 104 (18.9) | 214 (43.7) | < 0.001 |

Values are (n%) unless otherwise indicated.

Abbreviations: **ASA,** American society of Anesthesiologists; **CA19-9,** carbohydrate antigen 19-9; **SII,** systemic immune-inflammatory index; **TLNE,** the total number of lymph nodes examined; **PI/MF+PI,** periductal infiltrating/ mass forming plus periductal infiltrating

**Supplementary Table 4**. Multivariable Cox regression analysis including an interaction term between SII and adjuvant chemotherapy for recurrence-free survival among patients with TLNE < 6.

| Variables | HR 95%CI | p-value |
| --- | --- | --- |
| Age | 0.99 [0.98, 1.00] | 0.041 |
| Sex, male  (ref: Female) | 1.33 [1.07, 1.64] | 0.009 |
| ASA classification, > 2  (ref: Classification 1,2) | 1.02 [0.76, 1.35] | 0.916 |
| Geographic region, Eastern countries  (ref: Western countries) | 1.26 [0.92, 1.73] | 0.152 |
| Year of surgery, 2011-2023  (ref: 2000-2010) | 0.90 [0.73, 1.12] | 0.340 |
| Neoadjuvant chemotherapy | 1.11 [0.71, 1.72] | 0.651 |
| Cirrhosis | 1.07 [0.81, 1.43] | 0.633 |
| CA19-9, > 54 U/ml  (ref: ≤ 54 U/ml) | 0.98 [0.74, 1.29] | 0.880 |
| CA19-9, Unknown  (ref: ≤ 54 U/ml) | 1.04 [0.82, 1.32] | 0.744 |
| Tumor size, > 5cm  (ref: ≤ 5cm) | 1.54 [1.24, 1.91] | < 0.001 |
| Multiple lesion  (ref: single lesion) | 1.69 [1.26, 2.29] | 0.001 |
| Lymph node dissection | 1.05 [0.84, 1.32] | 0.644 |
| Pathological T category, T3/4  (ref: T1/2) | 1.15 [0.88, 1.51] | 0.310 |
| Major vascular invasion | 1.14 [0.82, 1.60] | 0.432 |
| Microvascular invasion | 1.25 [0.96, 1.63] | 0.097 |
| Morphologic type, PI/MF+PI  (ref: MF/IG) | 1.32 [0.96, 1.83] | 0.089 |
| Grade, poor/undifferentiated,  (ref: well/moderate) | 1.14 [0.86, 1.51] | 0.364 |
| Perineural invasion | 1.38 [1.03, 1.84] | 0.032 |
| Adjuvant chemotherapy (SII Low [< 738.4]) | 1.00 [0.74, 1.37] | 0.977 |
| Adjuvant chemotherapy (SII High [≥ 738.4]) | 1.14 [0.81, 1.59] | 0.460 |
| SII, High (≥ 738.4) (Adjuvant chemotherapy [-]) | 1.32 [1.02, 1.71] | 0.036 |
| SII, High (≥ 738.4) (Adjuvant chemotherapy [+]) | 1.49 [1.07, 2.09] | 0.020 |
| Adjuvant chemotherapy × SII | 1.13 [0.74, 1.72] | 0.568 |

Abbreviations: **ASA,** American society of Anesthesiologists; **CA19-9,** carbohydrate antigen 19-9; **SII,** systemic immune-inflammatory index; **MF,** mass-forming; **IG,** intraductal growth; **PI/MF+PI,** periductal infiltrating/ mass forming plus periductal infiltrating
